# Ferritinophagy Is Involved in the Advanced Glycation End Products‐Induced Ferroptosis of Vascular Endothelial Cells in Diabetes Mellitus

**DOI:** 10.1111/jcmm.70857

**Published:** 2025-10-26

**Authors:** Yi Huang, Yongzhi Jin, Junsheng Hu, Mengfan Li, Ming Tian, Guang Zeng, Rong Huang

**Affiliations:** ^1^ Department of General Surgery Putuo Hospital Shanghai University of Traditional Chinese Medicine Shanghai China; ^2^ Department of General Surgery LiQun Hospital, Putuo District Shanghai China; ^3^ Department of Burn Ruijin Hospital, Shanghai Jiaotong University School of Medicine Shanghai China

**Keywords:** advanced glycation end products, diabetes mellitus, ferritinophagy, ferroptosis, vascular endothelial cells

## Abstract

This study investigated the role of advanced glycation end products (AGEs) and ferritinophagy‐dependent ferroptosis in the pathogenesis of diabetic atherosclerosis obliterans. We investigated the effects of AGEs on ferroptosis and ferritinophagy in HUVECs. The methods employed included the CCK‐8 assay, reactive oxygen species (ROS) determination, Fe^2+^ measurement, Western blot, quantitative PCR (qPCR), transmission electron microscopy (TEM) and immunofluorescence. Additionally, we established type 2 diabetes rat models and atherosclerosis obliterans (ASO) models. Blood flow in the rats' lower limbs was measured using the RFLSI III Laser Speckle Imaging System. Furthermore, levels of glutathione (GSH) and malondialdehyde (MDA) were measured to evaluate oxidative stress and lipid peroxidation. Upon exposure of HUVECs to AGEs, a reduction in cell viability was observed, accompanied by a significant increase in iron ion and MDA levels, and a notable decrease in GSH levels, indicating the induction of ferroptosis. The addition of ferrostatin‐1 to AGEs‐treated HUVECs mitigated ferroptosis. TEM, immunofluorescence and Western blot analyses confirmed that AGEs can induce ferritinophagy in HUVECs. Furthermore, compared to the human serum albumin (HSA) group, the AGEs‐treated group exhibited significantly elevated levels of reactive oxygen species (ROS) and MDA, along with a marked reduction in GSH levels. These alterations were reversed by the addition of MitoTEMPOL to the AGEs group. Animal experiments further confirmed that the clearance of intracellular ROS alleviated ferritinophagy‐dependent ferroptosis. This study provides an insight into therapeutic strategies for alleviating ASO in DM by interfering with the ferritinophagy‐dependent ferroptosis due to AGEs accumulation.

## 1 Introduction

Atherosclerosis obliterans (ASO) of the lower extremities has a high incidence in diabetic patients, with endothelial cell damage and subsequent pathological changes playing a critical role in this process [1]. Type 2 diabetes mellitus (T2DM), characterised by hyperglycaemia, is a multifactorial metabolic disorder projected to affect 693 million adults globally by 2045 [2]. Microvascular and macrovascular complications of diabetes are the leading causes of mortality and disability in diabetic patients [3]. In a hyperglycaemic state, non‐enzymatic glycation reactions accelerate, leading to the formation of advanced glycation end products (AGEs) [4]. AGEs bind to various cell surface receptors, particularly the receptor for advanced glycation end products (RAGE), activating signalling pathways such as MAPK/ERK, TGF‐β, JNK and NF‐κB, which exacerbate oxidative stress and inflammation [5]. The activation of the AGEs/RAGE axis not only affects insulin signalling and metabolic homeostasis but also causes damage to vascular endothelial cells by inducing oxidative stress and inflammatory responses, which are key mechanisms in the pathogenesis of diabetic ASO [6].

AGEs can accumulate through endogenous and exogenous pathways. Endogenous AGEs are primarily formed through the Maillard reaction, which is accelerated under hyperglycaemic conditions [7]. Exogenous AGEs are mainly found in foods processed at high temperatures [8]. The accumulation of AGEs leads to long‐term cellular dysfunction, a phenomenon known as ‘metabolic memory’ [9]. In diabetic patients, the accumulation of AGEs is closely associated with various complications, including cardiovascular disease, kidney disease, retinopathy and neuropathy [10].

Ferroptosis, a form of programmed cell death distinct from apoptosis, necrosis and autophagy, has been found to be associated with diabetes and its complications, such as cardiomyopathy, nephropathy and retinopathy [11]. Ferroptosis involves various cellular metabolic pathways, including iron metabolism, mitochondrial activity, redox homeostasis and multiple disease‐related signalling pathways [12]. Although the role of ferroptosis in diabetes and its complications has been studied, its specific role in ASO has not been fully explored. Understanding the potential mechanisms and regulatory networks of ferroptosis and ferritinophagy may provide effective strategies for treating diabetic ASO [13].

Ferritinophagy is the process of regulating intracellular iron metabolism by degrading ferritin. Ferritinophagy, mediated by nuclear receptor coactivator 4 (NCOA4), transports ferritin to autolysosomes for degradation, releasing free iron, which may induce ferroptosis [14]. Based on this, we hypothesise that AGEs can induce the accumulation of ROS and oxidative stress, leading to endothelial cell ferroptosis, causing endothelial cell damage and thereby triggering atherosclerosis in diabetic patients.

This experiment aims to study the expression of ferroptosis‐related genes in diabetic ASO patients, cell models and animal experiments, and to explore the role of AGEs in vascular endothelial damage. Researchers used HUVECs (human umbilical vein endothelial cells) for experiments, employing methods such as CCK‐8 assay, ROS measurement, Fe^2+^ (iron ion) measurement, scratch and tube formation assays, and transcriptome analysis to study the effects of AGEs on HUVECs and the mechanism of AGE‐induced ferroptosis.

## 2 Methods

### 2.1 Clinical Sample Collection

Blood vessel samples were collected from ten healthy controls and ten diabetic patients at Putuo District Central Hospital. The normal control samples were obtained from amputated specimens of patients with no history of diabetes, while the diabetic samples were derived from amputated specimens of diabetic patients with atherosclerotic occlusive disease (ASO). Written informed consent was obtained from all patients or their relatives. The retrieval of blood vessel samples was approved by the Ethics Committee of Putuo District Central Hospital, Shanghai (Ethics Approval No. PTEC‐R‐2021‐13‐1), and was conducted in accordance with established laboratory protocols.

### 2.2 Detection of AGEs

After homogenising the vascular tissue and quantifying the protein, equal amounts of the protein supernatant were taken and assessed using a fluorescence spectrometer with excitation at 370 nm and emission at 440 nm.

### 2.3 Haematoxylin and Eosin (H&E) Staining

The vascular tissues were fixed in 4% paraformaldehyde (E672002, Sangon, China) for 48 h and subsequently embedded in paraffin. Longitudinal sections of 5 μm thickness were then cut and stained with haematoxylin and eosin to assess the pathological changes in the vascular tissue. These slides were analysed using an optical microscope.

### 2.4 Immunofluorescence

Vascular tissues and cells were fixed in 4% paraformaldehyde (PFA) at room temperature for 10 to 15 min and then permeabilised with 0.1% Triton X‐100 in phosphate‐buffered saline (PBS) for 30 min at room temperature. Following permeabilisation, the tissues were blocked with 1% bovine serum albumin (BSA) in PBS for 1 h at room temperature. Subsequently, the tissues were incubated overnight at 4°C with primary antibodies against CD8 (1:100, Proteintech, USA) and Granzyme B (Cell Signalling Technology, USA). After washing with PBS, the tissues were incubated with Alexa Fluor 488 and 594 secondary antibodies (Proteintech, USA) for 2 h at room temperature. The nuclei were stained with DAPI. Images were captured using a Confocal Laser Scanning Microscope (Leica TCS SP5, Germany).

### 2.5 Quantitative Real‐Time Polymerase Chain Reaction (qPCR)

Total RNA was extracted from HUVEC cells using TRIzol reagent (15,596,018, Thermo Fisher Scientific) and subsequently reverse transcribed into cDNA using the PrimeScript RT Reagent Kit with gDNA Eraser (RR047Q, Takara, Japan) according to the manufacturer's instructions. Quantitative PCR (qPCR) was performed on a C1000 Touch Real‐Time PCR System (Bio‐Rad) utilising Takara's SYBR Premix Ex Taq (RR420Q, Takara, Japan). The thermocycling conditions included an initial hold at 95°C for 30 s, followed by 40 cycles of 95°C for 5 s and 60°C for 34 s. GAPDH was used as the internal reference gene, and the primer sequences are listed in Table 1. Relative gene expression levels were calculated using the 2^−ΔΔCt^ method.

**TABLE 1:** The primer sequences used in this study.

GAPDH	F	GTCTCCTCTGACTTCAACAGCG
	R	ACCACCCTGTTGCTGTAGCCAA
ACSL4	F	GCTATCTCCTCAGACACACCGA
	R	AGGTGCTCCAACTCTGCCAGTA
GPX4	F	ACAAGAACGGCTGCGTGGTGAA
	R	GCCACACACTTGTGGAGCTAGA
FTH1	F	TGAAGCTGCAGAACCAACGAGG
	R	GCACACTCCATTGCATTCAGCC
ATP5B	F	TCATGCTGAGGCTCCAGAGTTC
	R	ACAGTCTTGCCAACTCCAGCAC
NCOA4	F	GCTTGCTATTGGTGGAGTTCTCC
	R	GCCATACCTCACGGCTTCTAAG

### 2.6 Western Blotting

Total proteins were extracted using RIPA lysis buffer (R0020, Solarbio, China). Protein concentration was subsequently quantified using the BCA protein assay kit (C503051, Sangon, China). A total of 30 μg of protein samples from each group were subjected to electrophoresis on 10% SDS‐PAGE gels, followed by transfer to nitrocellulose membranes (F619511, Sangon, China). The membranes were blocked with 5% skim milk in TBST at room temperature for 1 h, after which they were washed and incubated with the primary antibodies (Anti‐ACSL4 (22401–1‐AP, Proteintech), Anti‐GPX4 (67763–1‐Ig, Proteintech), Anti‐FTH1 (10727–1‐AP, Proteintech), Anti‐LC3B (18725–1‐AP, Proteintech) and Anti‐NCOA4 (DF4255, Proteintech)) overnight at 4°C. Following washes with TBST, the membranes were incubated with corresponding HRP‐conjugated secondary antibodies at room temperature for 2 h. The target bands were subsequently visualised using a chemiluminescent ECL kit (D601039, Sangon, China) and detected with a chemiluminescent imaging system (LAS‐4000, GE, USA).

### 2.7 Preparation and Identification of AGEs

The preparation and detection methods for AGEs have been documented in our prior research [15]. AGEs were stored at −20°C until they were utilised.

### 2.8 Cell Culture and Treatment

The human umbilical vein endothelial cells (HUVEC) were obtained from iCell Bioscience Inc. (Shanghai, China). The cells were cultured in DMEM supplemented with 10% foetal bovine serum (FBS) at 37°C in a humidified atmosphere containing 5% CO₂. Advanced glycation end products (AGEs), Ferrostatin‐1 (HY‐100579, MCE), Z‐VAD‐FMK (HY‐16658B, MCE), Bafilomycin A1 (HY‐100558, MCE) and MitoTEMPOL (HY‐112879, MCE) were dissolved in dimethyl sulfoxide (DMSO, Sigma). Subsequently, human umbilical vein endothelial cells (HUVECs) were pretreated with various agents, including human serum albumin (HSA), AGEs, Ferrostatin‐1, Z‐VAD‐FMK, Bafilomycin A1 and MitoTEMPOL, for 1 h prior to conducting different assays. Additionally, HUVECs were co‐cultured with varying concentrations of AGEs (0, 10, 20, 40 and 80 μM) for 48 h. Furthermore, HUVECs were exposed to a fixed concentration of AGEs (20 μM) for different durations (0, 24, 48 and 72 h).

### 2.9 Cell Viability Assay

HUVEC cells were seeded into 96‐well plates at a density of 2 × 10 ^ 3 cells per well overnight. Following various treatments, 10 μL of CCK‐8 solution was added to each well and incubated for 2 h at 37°C. The absorbance at 450 nm was measured using a Multiskan FC spectrophotometer (Thermo, USA).

### 2.10 Determination of Iron Ion, Glutathione (GSH) and Hippocampal Malondialdehyde (MDA) Levels

According to the manufacturer's instructions, the levels of iron ion, GSH and MDA in cells from different treatment groups were quantified using the Iron Assay Kit (bc4355, Solarbio), GSH Assay Kit (bc1175, Solarbio) and MDA Assay Kit (bc0025, Solarbio), respectively. The absorbance values of iron ion, MDA and GSH were measured using a microplate reader at 520 nm, 420 nm and 530 nm, respectively.

### 2.11 Flow Cytometry

Following the manufacturer's instructions, cells were incubated with 10 μM C11‐BODIPY581/591 (Thermo Fisher Scientific) for 30 min at 37°C. Subsequently, the cells were washed with PBS, trypsinized, collected and analysed using flow cytometry.

### 2.12 Transmission Electron Microscopy

Cells were harvested and fixed in 2.5% glutaraldehyde (A17876, Alfa Aesar, USA), followed by post‐fixation in 1% osmium tetroxide. After fixation, the cells underwent dehydration through a graded ethanol series. Subsequently, the samples were embedded in epoxy and acrylic resins. Ultrathin sections, approximately 1 μm thick, were cut using an ultramicrotome equipped with glass knives. These sections were then stained with sodium acetate‐lead citrate. Finally, the thin sections of each sample were examined using a transmission electron microscope (Hitachi H‐7500, Japan).

### 2.13 Mitochondrial Membrane Potential Assay

After various treatments, cells were harvested and incubated with 10 μM TMRE working solution (MedChemExpress, HY‐D0985A) at 37°C for 30 min. After washing with PBS, the fluorescence intensity was measured at excitation and emission wavelengths of 530 nm and 590 nm, respectively.

### 2.14 Cellular Mitochondrial ROS

After various treatments, cells were harvested and incubated with 5 μM MitoSOX Red (Thermo Fisher Scientific, M36008) working solution at 37°C for 30 min. After washing with PBS, the fluorescence microscopy was used for observation and imaging.

### 2.15 Measurement of Intracellular ROS Level

The fluorescent probe DCFH‐DA (Sigma‐Aldrich, United States) was employed to assess intracellular ROS levels according to the manufacturer's instructions. Cells were seeded into 6‐well plates at a density of 2 × 10^5^ cells per well and subjected to various treatments. Subsequently, the cells were incubated with the DCFH‐DA probe at 37°C for 30 min in the dark. After three washes with serum‐free medium, the fluorescence of the cells was captured using an Axio Observer D1 fluorescence inverted microscope (ZEISS, Germany) and quantified with a BD FACS Canto II flow cytometer (BD Biosciences, United States).

### 2.16 Animals

Eighteen Sprague Dawley male rats (weighing 240–2770 g) were obtained from the Shanghai Slack Laboratory Animal Co. Ltd. The rats were maintained under standard laboratory conditions, with a humidity of 40%–50%, a 12‐h light/dark cycle and a temperature range of 20°C–24°C. Prior to the injection of streptozotocin (STZ, Sigma, USA), the animals were fasted for 24 h. Diabetes was induced by administering a single intraperitoneal injection of STZ solution (65 mg/kg) dissolved in 0.1 M citrate buffer (pH 4.2). Rats with blood glucose levels of ≥ 11.2 mmol/L were selected and classified as diabetic. To manage severe diabetic symptoms, protamine zinc insulin was administered intraperitoneally at a dose of 1–4 U/day to maintain blood glucose levels at 16.7 mmol/L, thereby reducing mortality. The experimental rats were housed in standard cages at the Department of Laboratory Animal Science, Fudan University, and were provided with standard pellet feed and unrestricted access to food and water, under natural light/dark conditions. The room was well ventilated, with ammonia levels kept below 20 pmol/L, relative humidity between 40% and 70% and a temperature maintained at 18°C–22°C.

Twelve diabetic rats were anaesthetised via intraperitoneal injection of 0.03 mL/kg of 10% chloral hydrate. The rats were then secured on the surgical table. Both hind limbs of each rat were disinfected, and a longitudinal incision was made from the midpoint of the groin to below the knee to expose the femoral artery up to the bifurcation of the popliteal artery. The femoral artery was clamped proximally and distally over a length of 1.5–2.0 cm using small arterial clamps. A 0.1 mL syringe fitted with a specially designed glass puncture needle was used to puncture the artery along its longitudinal axis from the proximal end. Sterile distilled water (0.2–0.3 mL) was slowly injected into the occluded section of the vessel until it was fully filled. After 5 min, the needle and arterial clamps were removed, and haemostasis and suturing were performed. The rats were then randomly divided into two groups: the model group (*n* = 6) and the ROS scavenger group (*n* = 6). The model group received a 33:67 (vol/vol) mixture of DMSO and normal saline, while the ROS scavenger group was administered XJB‐5‐131 at a dosage of 2 μmol/kg. Both groups were fed a high‐fat diet, which consisted of 62.8% standard feed, 20% lard, 150 g/L cholesterol, 20 g/L sodium cholate and 2 g/L propylthiouracil. After 8 weeks on the high‐fat diet, blood flow in the rats' lower limbs was measured using the RFLSI III Laser Speckle Imaging System (RWD Life Science, Shenzhen, China).

### 2.17 Statistical Analysis

Statistical analyses were performed using GraphPad Prism version 9.0. Results are presented as mean ± standard deviation (SD). Comparative analysis between two distinct groups was performed using the Student's *t*‐test. One‐way analysis of variance (ANOVA) was used to discern variances among multiple groups. Statistical significance was set at *p* < 0.05.

## 3 Results

### 3.1 AGEs Are Significantly Elevated in Diabetic Patients, and Both Ferroptosis and Ferritinophagy Are Concurrently Present in the Vascular Tissues of DM Patients

All subjects in the study were male. The normal control group ranged in age from 29 to 59 years, while the diabetic (DM) group ranged from 62 – 82 years. The DM group had a history of diabetes spanning 10 – 30 years and exhibited HbA1C levels between 8.9% and 11.7%.

**FIGURE 1:**
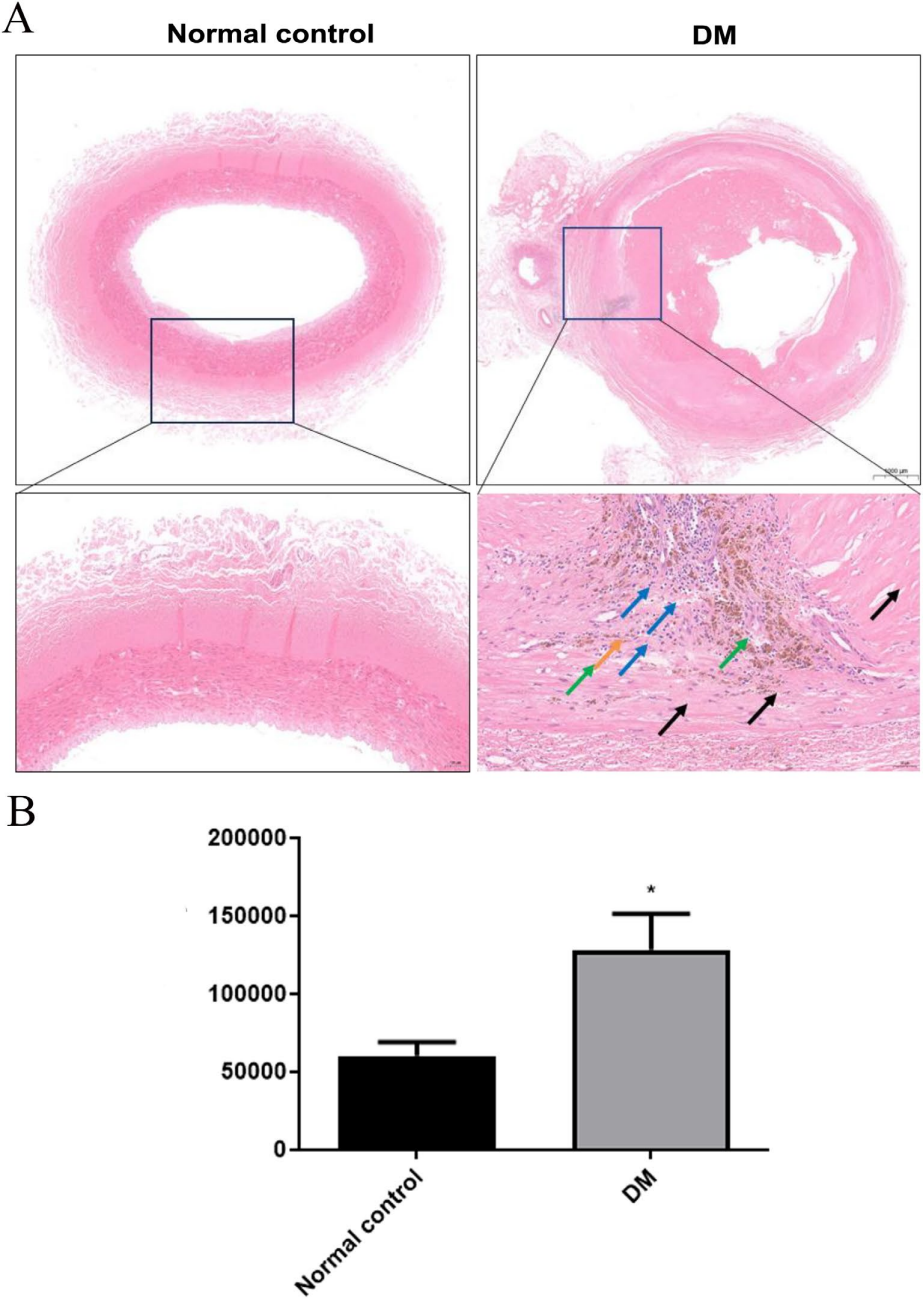
A. Representative images of H&E staining. B. The content of AGEs of the vascular cells in the normal control and diabetic groups. *p < 0.05.

HE revealed that in the DM patients, the vascular wall structure was unclear, with widespread absence of endothelial cells in the intima. The internal elastic membrane was irregularly arranged and disrupted in multiple regions. Additionally, the intima and media were largely replaced by proliferative connective tissue and collagen fibres, and there was significant localised lymphocyte infiltration Figure 1A. Figure 1B shows that compared to the normal control group, the content of AGEs in the vascular tissues of the DM group was significantly elevated.

**FIGURE 2:**
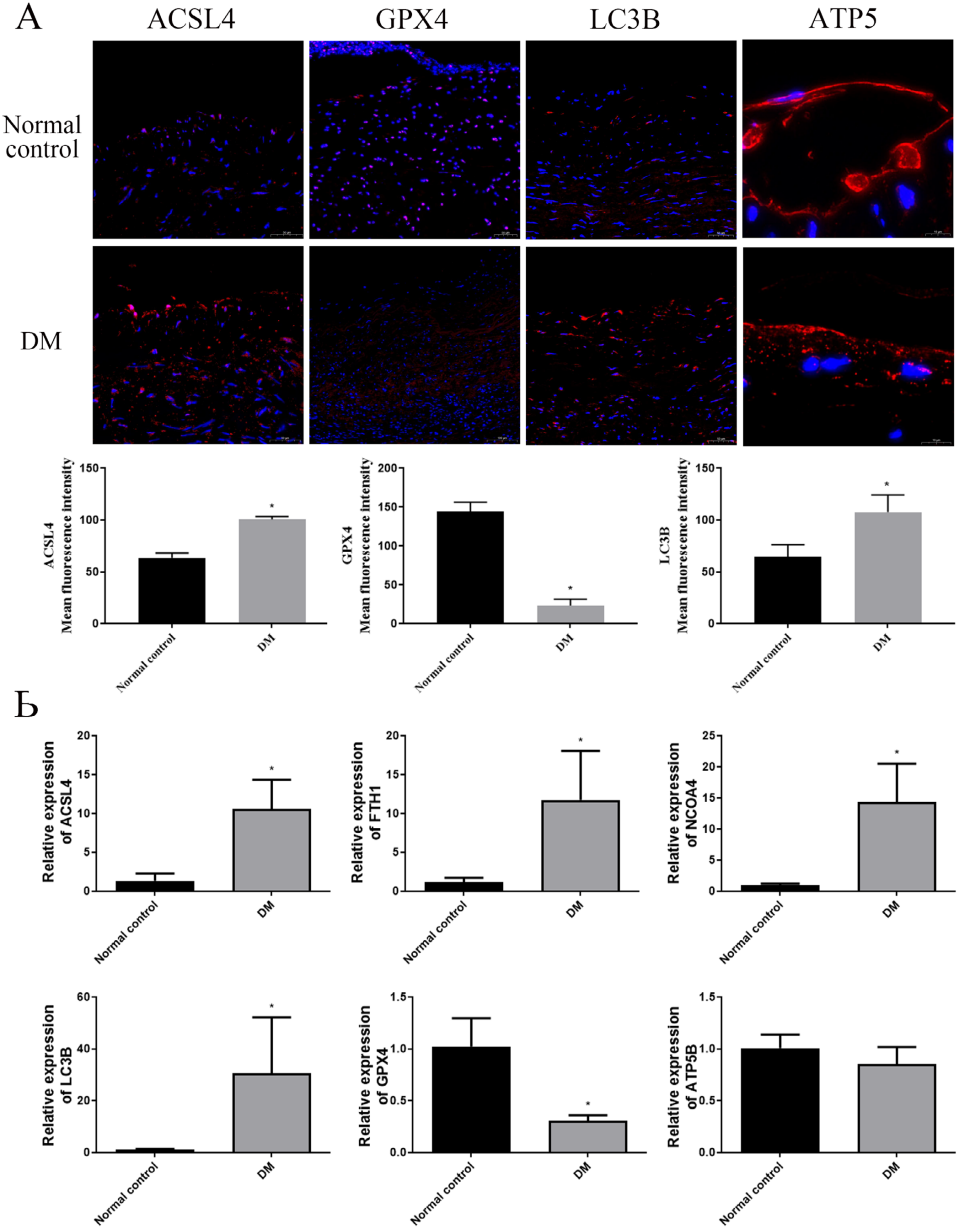
A. Immunofluorescence detection of the ferroptosis‐related gene expressions in the vascular tissue samples. B. The mRNA ACSL4, FTH1, NCOA4, LC3B, GPX4 and ATP5B expression levels were detected using qPCR. Data were expressed as mean ± S.D. *p < 0.05.

We further investigated genes related to ferroptosis and ferritinophagy. Immunofluorescence analysis demonstrated that, compared to the control group, the expression of ACSL4 and LC3B was increased, whereas the expression of GPX was decreased in the DM group (*p* < 0.05, Figure 2A). In the control group, ATP5B exhibited a filamentous/strip‐like structure, while in the DM group, it appeared as dots/clusters, indicating mitochondrial damage (Figure 2A). The qPCR results also showed that the expression levels of ACSL4, NCOA4, LC3B and FTH1 were significantly elevated in the DM group, while the expression of GPX4 was significantly decreased (*p* < 0.05, Figure 2B). However, there was no significant difference in ATP5B expression between the two groups (*p* > 0.05, Figure 2B). These findings suggest the concurrent presence of ferroptosis and ferritinophagy in the vascular tissues of DM patients.

### 3.2 AGEs Induce Ferroptosis in HUVEC Cells

**FIGURE 3:**
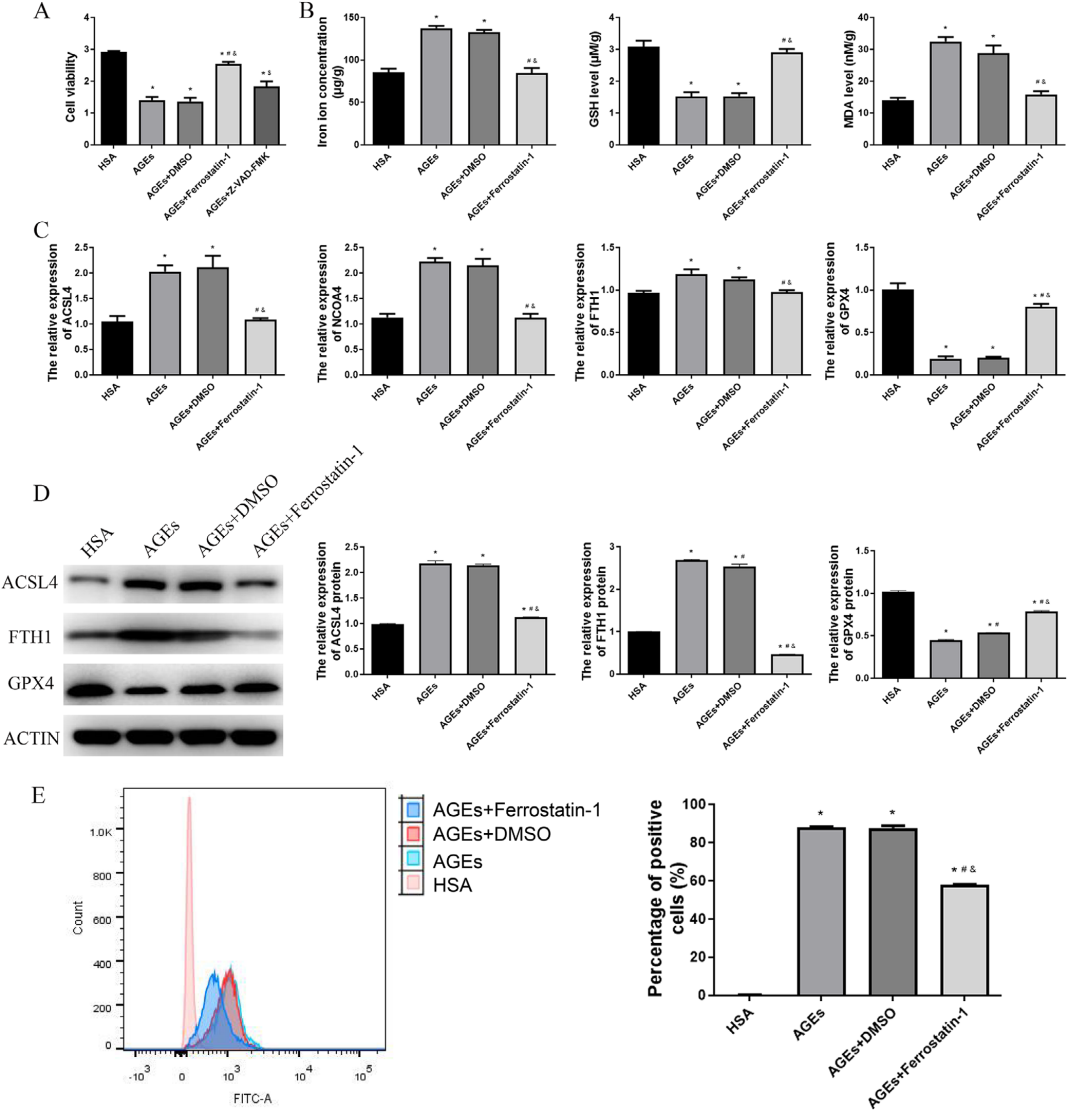
AGEs induce ferroptosis in HUVEC cells. A. Cell viability of HUVEC cells was detected by a CCK8 assay. The levels of iron ions (B), GSH (C) and MDA (D) in the HUVEC cells were determined using commercial kits. E. The mRNA ACSL4, NCOA4, FTH1 and GPX4 expression levels were detected using qPCR. F. The protein ACSL4, FTH1 and GPX4 expression levels were detected using western blot. G. The lipid peroxidation in HUVEC cells was detected using flow cytometry with the C11‐BODIPY581/591 fluorescent probe. *p < 0.05, compared with HAS group; ^#^ *p* < 0.05, compared with AGEs group; ^&^ *p* < 0.05, compared with AGEs+DMSO group.

To further investigate the occurrence of ferroptosis in the vasculature of diabetic patients, we conducted cellular experiments using HUVEC cells and induced them with AGEs. The CCK‐8 assay results indicated that cell viability was significantly reduced in the AGEs group compared to the HSA group (*p* < 0.05, Figure 3A). Notably, the addition of a ferroptosis inhibitor (AGEs + Ferrostatin‐1 group) partially reversed the reduction in cell viability (*p* < 0.05, Figure 3A). However, there was no significant difference in cell viability between the AGEs + Z‐VAD‐FMK group and both the AGEs and AGEs + DMSO groups (*p* > 0.05, Figure 3A). These results indicate that there is ferroptosis rather than apoptosis in HUVECs induced by AGEs.

Subsequently, we evaluated the levels of iron ions, GSH and MDA in cells across various treatment groups. Our findings revealed that, compared to the HSA group, the AGEs group exhibited significantly elevated levels of iron ions and MDA, along with a marked reduction in GSH levels (*p* < 0.05, Figure 3B). However, the addition of the ferroptosis inhibitor Ferrostatin‐1 to the AGEs group markedly decreased the concentrations of iron ions and MDA (*p* < 0.05, Figure 3B,C) while significantly increasing GSH levels (*p* < 0.05, Figure 3D). Next, the expression levels of ferroptosis‐related markers were assessed using qPCR and Western Blot. The results from both qPCR indicated that, compared to the HSA group, the expressions of ACSL4, NCOA4 and FTH1 were significantly elevated in the AGEs and AGEs+DMSO groups, while the expression level of GPX4 was markedly decreased (*p* < 0.05, Figure 3E). Furthermore, compared to the AGEs and AGEs+DMSO groups, the addition of a ferroptosis inhibitor resulted in a significant reduction in the expression levels of ACSL4, NCOA4 and FTH1, whereas the expression level of GPX4 showed a significant increase (*p* < 0.05, Figure 3E). The western blot showed that the result trend was similar to that of qPCR (Figure 3F). Moreover, we quantified lipid peroxidation in HUVEC cells using flow cytometry with the C11‐BODIPY581/591 fluorescent probe, a well‐established marker of ferroptosis. The results indicated that the AGEs group significantly increased lipid peroxidation levels in HUVEC cells compared to the HSA group (*p* < 0.05, Figure 3G). However, the addition of a ferroptosis inhibitor to the AGEs group effectively reduced the lipid peroxidation levels (*p* < 0.05, Figure 3G). These findings indicate that AGEs could induce ferroptosis in HUVEC cells.

### 3.3 AGEs Induce Ferritinophagy in HUVEC Cells

**FIGURE 4:**
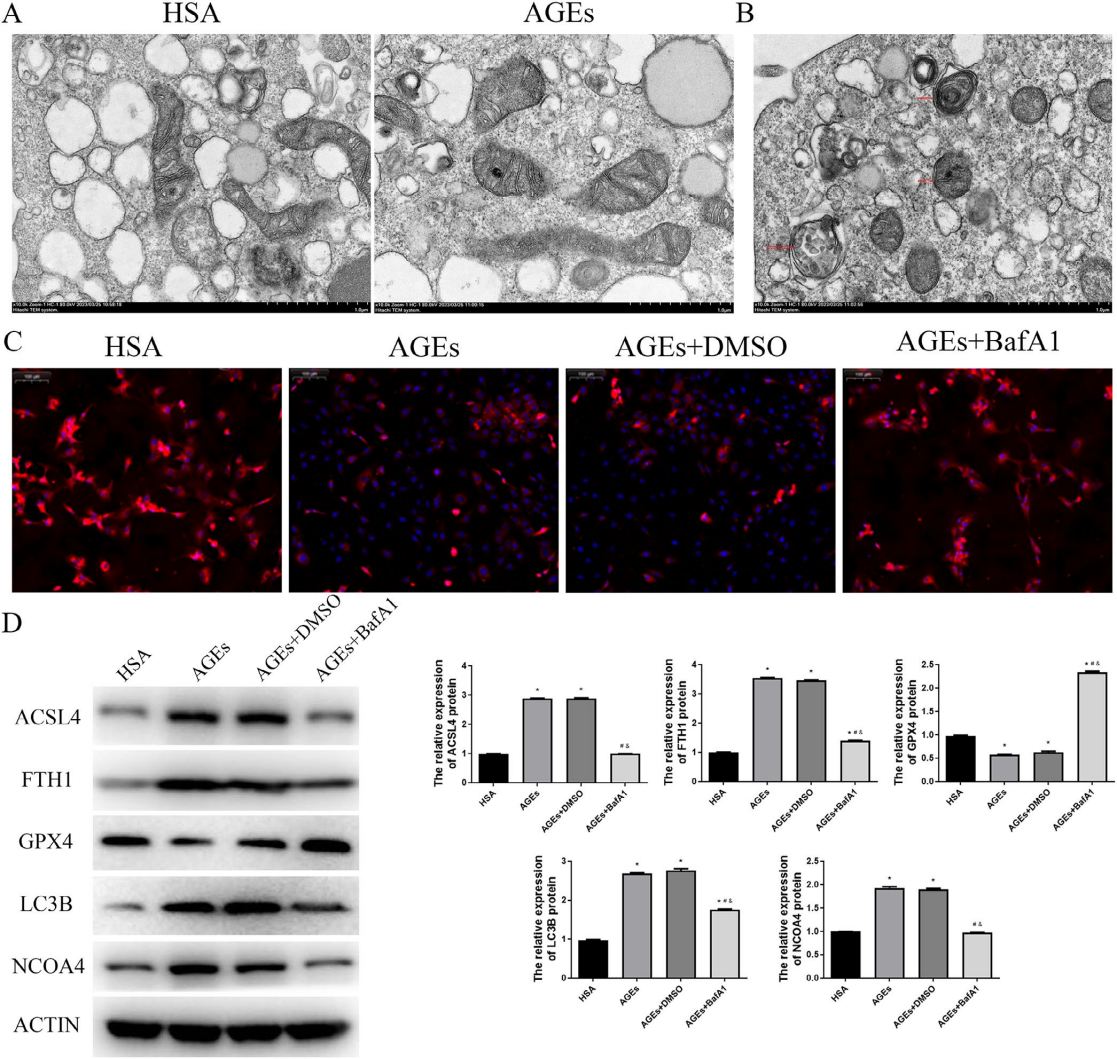
AGEs induce ferritinophagy in HUVEC cells. A. Electronic microscopic observation of mitochondrial morphology. B. Electronic microscopic observation of autophagosomes. The red arrows indicate the autophagosomes. C. ATP5B immunofluorescence detection. D. The protein ACSL4, FTH1 and GPX4 expression levels were detected using western blot. *p < 0.05, compared with HAS group; ^#^ *p* < 0.05, compared with AGEs group; ^&^ *p* < 0.05, compared with AGEs+DMSO group.

We also investigated whether AGEs could also induce autophagy (ferritinophagy) in HUVEC cells. Initially, we examined the morphological changes in HUVEC cells treated with AGEs using transmission electron microscopy (Figure 4A). The results indicated significant alterations in mitochondrial morphology in the AGEs group compared to the HSA group (Figure 4A). Mitochondria in AGEs‐treated cells exhibited pronounced swelling. Furthermore, the presence of autophagosomes within the cells suggests the occurrence of autophagy (Figure 4B). Next, we performed immunofluorescence analysis to assess the autophagy‐related protein ATP5B. The immunofluorescence results demonstrated that upon AGEs treatment, ATP5B within the cells transitioned from a filamentous to a punctate distribution (Figure 4C). Additionally, we conducted Western blot analysis to evaluate the levels of ferritinophagy‐related proteins. The results showed that, compared to the HSA group, the protein levels of ACSL4, FTH1, LC3B and NCOA4 were significantly elevated in both the AGEs and AGEs+DMSO groups, whereas the protein level of GPX4 was significantly reduced (*p* < 0.05, Figure 4D). However, the inclusion of autophagy inhibitors in the treatment with AGEs significantly inhibited the increase in protein levels of ACSL4, FTH1, LC3B and NCOA4, as well as prevented the decrease in GPX4 protein levels (*p* < 0.05, Figure 4D). These findings showed that AGEs can induce ferritinophagy in HUVEC cells. The inhibition of ferritinophagy can mitigate AGEs‐induced ferroptosis, indicating that AGEs‐induced ferroptosis is dependent on ferritinophagy.

### 3.4 AGEs Promotes Ferroptosis and Ferritinophagy in HUVEC Cells and Leads to Mitochondrial Dysfunction

**FIGURE 5:**
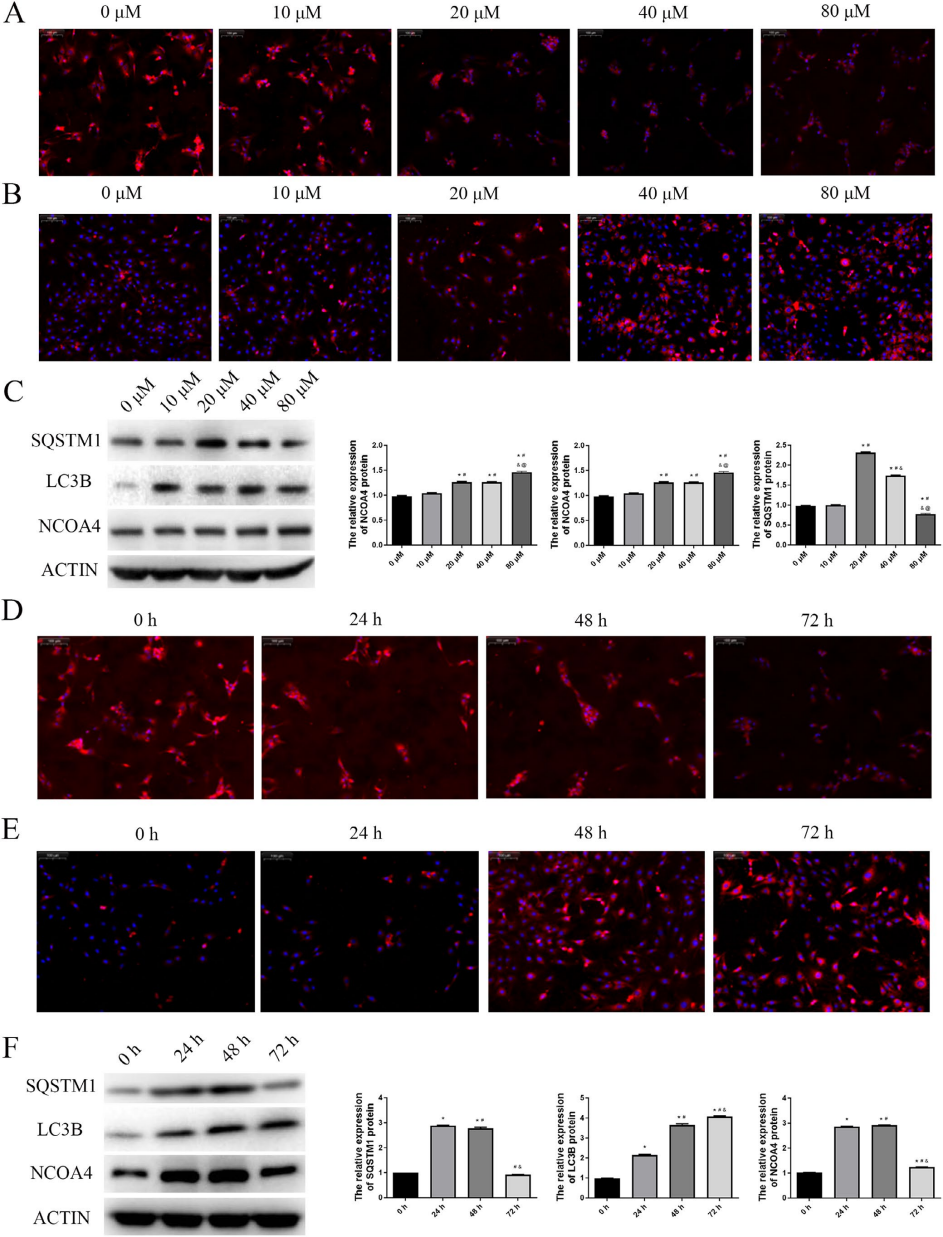
AGEs promote ferroptosis and ferritinophagy in HUVEC cells and lead to mitochondrial dysfunction. A. Representative image for TMRE labelling of HUVECs treated with different concentrations of AGEs. B. Representative image for MitoSOX labelling of HUVECs treated with different concentrations of AGEs. C. The protein SQSTM1, LC3B and NCOA4 expression levels were detected using western blot. *p < 0.05, compared with 0 μM AGEs group; ^#^ *p* < 0.05, compared with 10 μM AGEs group; ^&^ *p* < 0.05, compared with 20 μM AGEs group;^@^ *p* < 0.05, compared with 40 μM AGEs group. D. Representative image for TMRE labelling of HUVECs treated with 20 μM AGEs at 0, 24, 48 and 72 h. E. Representative image for MitoSOX labelling of HUVECs treated with 20 μM AGEs at 0, 24, 48 and 72 h. F. The protein SQSTM1, LC3B and NCOA4 expression levels were detected using western blot. *p < 0.05, compared with 20 μM AGEs at 0 h; ^#^ *p* < 0.05, compared with 20 μM AGEs at 24 h; ^&^ *p* < 0.05, compared with 20 μM AGEs at 48 h.

Given that ferroptosis is a form of reactive oxygen species (ROS)‐dependent regulated cell death and mitochondria are generally considered the primary sources of ROS production, we investigated whether mitochondria were damaged in HUVECs treated with varying concentrations of AGEs. The results demonstrated that as the concentration of AGEs increased, the fluorescence intensity of TMRE dye significantly decreased, indicating a substantial reduction in mitochondrial membrane potential and suggesting mitochondrial dysfunction and damage (Figure 5A). Concurrently, mitoSOX fluorescence intensity markedly increased with higher AGE concentrations, indicating a significant elevation in intracellular ROS levels (Figure 5B). We also conducted Western blot analysis on proteins related to ferroptosis and ferritinophagy, including SQSTM1, LC3B and NCOA4. The results indicated that AGEs significantly increase the expression of these ferroptosis and ferritinophagy‐associated proteins (Figure 5C).

Subsequently, we utilised a concentration of 20 μM for treatments at various time points (0, 24, 48 and 72 h). The results indicated that at 24 h, there was no significant difference in cellular phenotype compared to 0 h. However, after 48 h of treatment, the cells exhibited a significant decrease in mitochondrial membrane potential (Figure 5D) and an increase in mitochondrial ROS levels (Figure 5E). Additionally, we examined ferroptosis and ferritinophagy‐related proteins in cells treated for different durations and found that LC3B levels continued to increase with prolonged treatment, whereas SQSTM1 and NCOA4 showed peak expression at 48 h and decreased at 72 h (Figure 5F).

### 3.5 Scavenging ROS Mitigates Ferroptosis and Ferritinophagy Induced by AGEs in HUVECs

**FIGURE 6:**
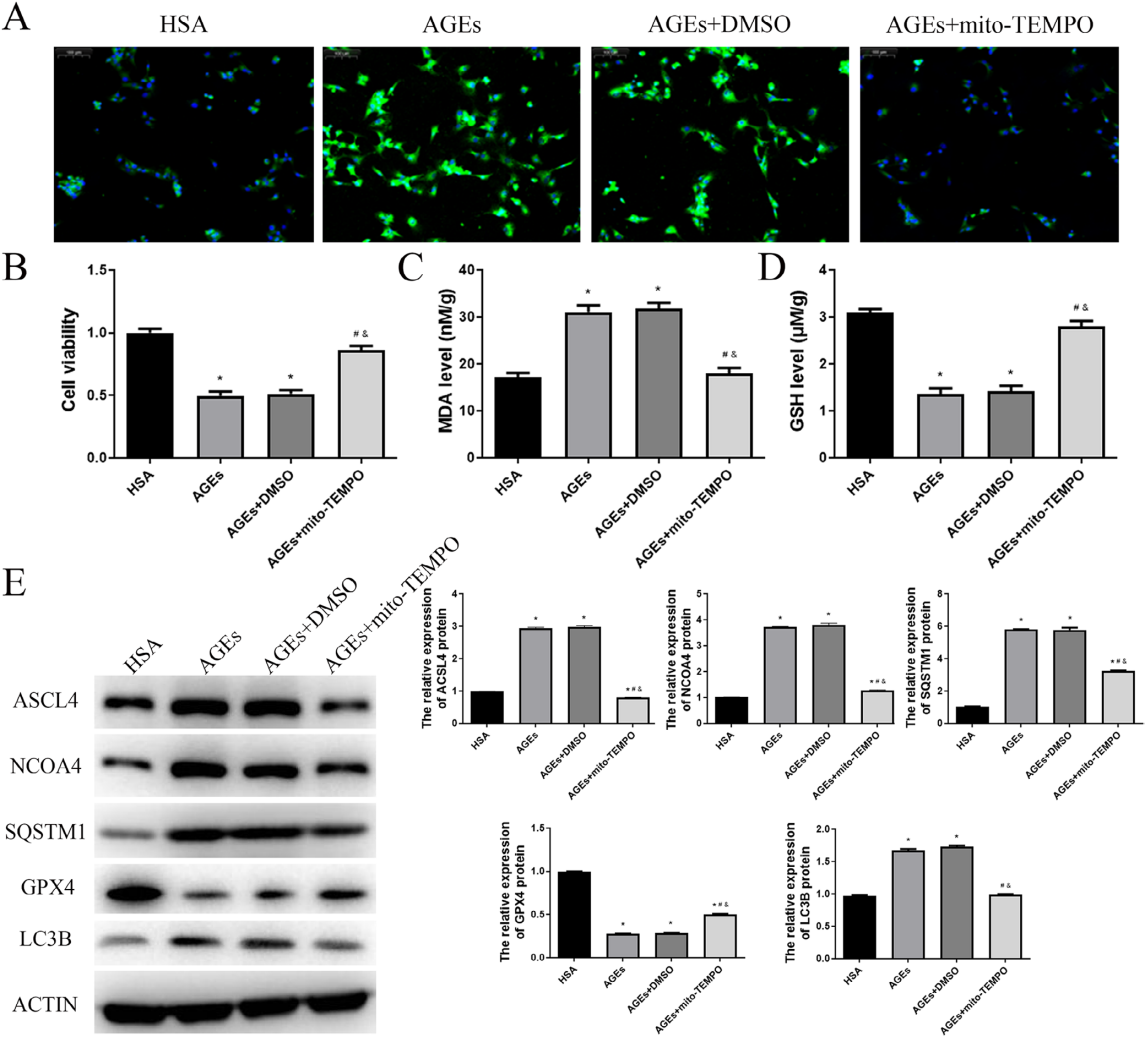
Scavenging ROS mitigates ferroptosis and ferritinophagy induced by AGEs in HUVECs. A. Effect of MitoTEMPOL on cellular ROS levels in HUVECs (fluorescence microscopy). B. Cell viability of HUVEC cells was detected by a CCK8 assay. The levels of MDA (C) and GSH (D) in the HUVEC cells were determined using commercial kits. The protein ACSL4, NCOA4, SQSTM1, GPX4 and LC3B expression levels were detected using western blot. *p < 0.05, compared with HAS group; ^#^ *p* < 0.05, compared with AGEs group; ^&^ *p* < 0.05, compared with AGEs+DMSO group.

To further investigate whether reducing ROS levels in HUVECs can mitigate AGEs‐induced ferroptosis and ferritinophagy, we employed the mitochondrial ROS scavenger MitoTEMPOL. Initially, fluorescence results indicated a significant increase in ROS levels in the AGEs and AGEs+DMSO groups compared to the HSA group (Figure 6A). However, the addition of MitoTEMPOL to the AGEs group significantly reduced intracellular ROS levels (Figure 6A). CCK‐8 assay results demonstrated that cell viability was significantly decreased in the AGEs and AGEs+DMSO groups compared to the HSA group, but this decrease was reversed upon the addition of MitoTEMPOL to the AGEs group (*p* < 0.05, Figure 6B). Subsequently, intracellular levels of MDA and GSH were measured. The results showed that MDA levels were significantly increased and GSH levels were significantly decreased in the AGEs group and the AGEs+DMSO group compared to the HSA group (*p* < 0.05, Figure 6C,D). These changes were reversed by the addition of MitoTEMPOL to the AGEs group (*p* < 0.05, Figure 6C,D). Additionally, western blot analysis was conducted to assess the expression of proteins related to ferroptosis and ferritinophagy. Compared to the HSA group, the protein levels of ACSL4, NCOA4, SQSTM1 and LC3B were significantly elevated in the AGEs and AGEs+DMSO groups, whereas the protein level of GPX4 was significantly reduced (*p* < 0.05, Figure 6E). The addition of MitoTEMPOL to the AGEs group significantly reversed these changes (*p* < 0.05, Figure 6E). These findings indicate that scavenging ROS mitigates ferroptosis and ferritinophagy induced by AGEs in HUVECs.

### 3.6 Functional Verification of Animal Experiments

**FIGURE 7:**
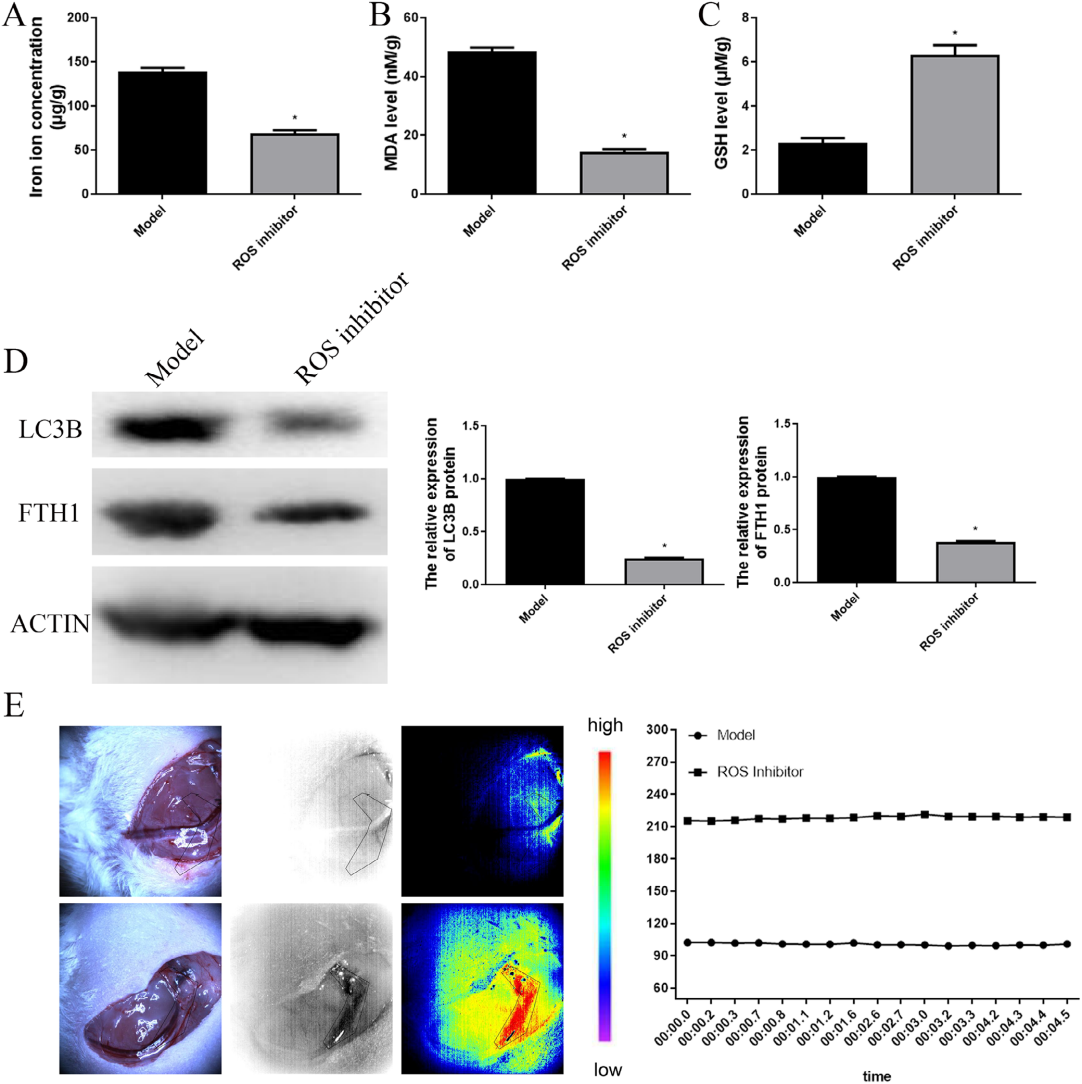
The levels of iron ions (A), GSH (B) and MDA (C) in the HUVEC cells were determined using commercial kits. D. The protein LC3B and FTH1 expression levels were detected using western blot. E. Representative images of the femoral artery blood flow monitoring in the groups. *p < 0.05.

Subsequently, we further investigated the impact of ROS clearance on ferroptosis and ferritinophagy induced by AGEs through a series of animal experiments. We established a diabetic ASO model and intervened with an ROS inhibitor. Our findings demonstrated that the ROS inhibitor significantly reduced the concentration of iron ions in vascular tissues (Figure 7A) and the concentration of MDA (Figure 7B), while increasing the concentration of GSH (Figure 7C). We also assessed the relevant proteins and found that the expression of FTH1 and LC3B significantly decreased following ROS inhibitor intervention (*p* < 0.05, Figure 7D). Moreover, animal models showed that continuous measurement of femoral artery blood flow for 5 s in both groups of rats revealed significantly higher blood flow in the ROS inhibitor group compared to the model group (*p* < 0.05, Figure 7E). These results suggest that the elimination of ROS can mitigate AGEs‐induced ferroptosis and ferritinophagy.

## 4 Discussion

Previous studies have established a strong correlation between vascular complications in diabetic patients and elevated levels of AGEs, known to induce oxidative stress and cellular damage [16]. While diabetic vascular complications are often linked to apoptosis and necrosis, the specific role of AGEs in triggering ferroptosis and ferritinophagy remains underexplored. In this study, we examined the expression of ferroptosis‐related genes in DM‐ASO patients and utilised cell models and animal experiments to assess the impact of AGEs on vascular endothelial injury. Interestingly, we found that AGEs induced vascular endothelial injury by causing ferritinophagy‐dependent ferroptosis, introducing a novel perspective on the mechanistic role of ferroptosis in diabetic ASO. This study provides new insights into the mechanisms underlying diabetic ASO and lays the foundation for future therapeutic strategies.

AGEs, formed through non‐enzymatic reactions between reducing sugars and proteins, lipids, or nucleic acids, are characteristic markers of DM [5]. By comparing vascular samples from healthy and diabetic subjects, we observed a significant increase in AGEs in the latter group. AGEs are typically produced via the Maillard reaction, involving Schiff base formation, Amadori rearrangement and glycoxidation. Hyperglycaemia accelerates AGE production through multiple pathways [17]. By disrupting redox homeostasis, AGEs induce oxidative stress, potentially leading to ferroptosis—a distinct form of cell death characterised by iron‐dependent phospholipid hydroperoxide accumulation [18]. To investigate the potential role of ferritinophagy‐dependent ferroptosis in DM patients, we analysed the expression of ferroptosis‐related genes, including ACSL4, LC3B, NCOA4 and FTH1, in vascular samples. Our findings revealed significantly elevated expression levels of ACSL4, LC3B, NCOA4 and FTH1, alongside a marked decrease in GPX4 expression in diabetic samples. These results suggest the coexistence of ferroptosis and ferritinophagy in the vasculature of diabetic patients.

Ferroptosis, a regulated form of cell death independent of caspases, is primarily triggered by intracellular lipid peroxidation and is intricately controlled by GPX4 [13]. This process disrupts iron, amino acid and lipid metabolism, leading to the accumulation of ROS and influencing disease progression [19, 20]. There is a well‐established connection between diabetic complications and ferroptosis. Hyperglycaemia can cause iron overload, which promotes ROS production and oxidative stress, ultimately resulting in ferroptosis [21, 22]. Previous studies have suggested a link between excessive body iron storage and an increased risk of type 2 diabetes (T2D) [23]. Recent Mendelian randomisation studies further support a causal relationship between elevated systemic iron levels and a heightened risk of developing T2D [24]. Additionally, the ferroptosis inducer erastin has been shown to trigger ferroptosis in MIN6 β cells and impair glucose‐stimulated insulin secretion in human islets, effects that are mitigated by ferrostatin‐1, a ferroptosis inhibitor [25, 26]. In our study, we explored the mechanisms by which AGEs induce ferroptosis. Through a series of experiments, we confirmed the pivotal role of ferroptosis in vascular tissues of diabetic patients and in HUVEC cells. Notably, treatment with Ferrostatin‐1 significantly restored cell viability after AGEs exposure, reduced levels of iron ions and MDA, and increased GSH levels. Moreover, qPCR and Western blot analyses revealed that AGEs treatment led to increased expression of ACSL4, NCOA4 and FTH1, while GPX4 expression was decreased; these changes were partially reversed by Ferrostatin‐1. AGEs also caused significant lipid peroxidation and a decline in mitochondrial membrane potential, effects that were effectively alleviated by Ferrostatin‐1. These findings underscore the critical role of ferroptosis in AGEs‐induced cellular damage.

Ferritinophagy, a selective autophagic process mediated by NCOA4, plays a critical role in regulating ferroptosis by modulating ROS production and maintaining intracellular iron homeostasis [27]. Recent evidence highlights its involvement in the progression of diabetic complications. For instance, renal biopsies from patients with diabetic nephropathy show increased transferrin expression and iron deposition in renal tubular epithelial cells compared to healthy controls [28]. Moreover, the Rev‐Erbs agonist SR9009 has been shown to alleviate myocardial ischaemia/reperfusion (I/R) injury by downregulating ferritinophagy/ferroptosis signalling in type‐2 diabetic rats [29]. Additionally, inhibiting DNA (cytosine‐5)‐methyltransferase 1 (DNMT‐1) can reduce ferroptosis in diabetic myocardial I/R injury by modulating ferritinophagy [30]. In our study, we discovered that AGEs can induce ferritinophagy in HUVECs, resulting in mitochondrial morphological changes and significant alterations in related protein levels. Notably, in our study, we also observed irregular changes in SQSTM1 expression, a key autophagic adapter protein [31], in AGEs‐treated HUVECs depending on AGEs concentration and stimulation duration. SQSTM1 showed peak expression at 48 h and decreased at 72 h. SQSTM1 is typically degraded upon successful autophagosome‐lysosome fusion [32]. We speculated this irregular change in SQSTM1 may reflect impaired autophagic flux during AGEs‐induced ferritinophagy. Mechanistically, AGEs‐induced ROS may damage lysosomal integrity or inhibit fusion‐related proteins [33], leading to incomplete degradation of SQSTM1 despite active autophagosome formation. Alternatively, AGEs could upregulate SQSTM1 transcription via activating Nrf2 or NF‐κB signalling under oxidative stress [34, 35], further contributing to its transcriptional upregulation. This result indicated a dynamic expression change of SQSTM1 after activation of autophagy. Our result is consistent with a previous study, which showed that the expression level of SQSTM1 does not always inversely correlate with autophagic activity [36]. Importantly, accumulated SQSTM1 may enhance ferritinophagy by promoting the recruitment of ferritin to autophagosomes or exacerbate ferroptosis through disrupting iron homeostasis or redox balance, thereby amplifying AGEs‐induced endothelial damage.

In diabetic patients, elevated levels of AGEs accumulate and may serve as key inducers of ferroptosis [37]. AGEs increase intracellular ROS levels, triggering oxidative stress and promoting ferroptosis, characterised by lipid peroxidation and iron‐dependent cell death, with key molecular markers including ACSL4, GPX4 and NCOA4 [38, 39, 40]. By inducing ferritinophagy, AGEs elevate free iron ion concentrations, further exacerbating lipid peroxidation and cellular damage. ROS play a pivotal role in ferroptosis, as excessive ROS can damage mitochondria, reduce mitochondrial membrane potential and increase the risk of apoptosis and necrosis [41]. Ferroptosis inhibitors such as Ferrostatin‐1 mitigate ferroptosis by reducing lipid peroxidation and modulating the expression of related genes. ROS inhibitors alleviate oxidative stress and cellular damage induced by AGEs by lowering ROS levels. Our research indicates that AGEs exacerbate diabetes‐related vascular damage by inducing ferroptosis and ferritinophagy. Intervening in these processes with ROS scavengers and ferroptosis inhibitors may offer novel therapeutic strategies for diabetic vascular complications. These findings provide new insights into the pathological mechanisms of diabetic vascular lesions and suggest potential targets for therapies aimed at ferroptosis.

This study makes several pivotal contributions to our understanding of vascular damage in diabetes. First, we observed significant pathological changes in the vascular tissues of diabetic patients, such as endothelial cell loss and connective tissue replacement, highlighting the profound impact of diabetes on vascular structure. By simulating AGEs‐induced ferroptosis in HUVECs, we demonstrated the effects of AGEs on cell viability, iron ion concentration, lipid peroxidation and mitochondrial function, as well as the protective role of Ferrostatin‐1. Furthermore, the application of ROS scavengers in an ASO model showed potential in improving blood flow and reducing oxidative stress. Based on these findings, future research should explore the specific signalling pathways through which AGEs induce ferroptosis and ferritinophagy to better understand the molecular mechanisms involved. Developing and optimising therapeutic strategies targeting AGEs‐induced ferroptosis, particularly using ROS scavengers, is crucial given their efficacy in animal models. Additionally, studies should include diverse populations, encompassing women and various age groups, to verify the generalisability of the results. Long‐term follow‐up studies are necessary to assess the impact of AGEs and ferroptosis on vascular health in diabetic patients over time. Lastly, investigating the combined effects of Ferrostatin‐1 with other antioxidants or antidiabetic drugs could enhance therapeutic outcomes. Our study shows mitochondrial swelling upon AGEs stimulation in HUVECs, which might reflect the context‐dependent variability of mitochondrial morphological changes under ferroptotic stimuli, particularly in the context of AGEs‐induced oxidative stress and early‐stage ferroptosis. Mitochondrial morphology is dynamically regulated by the balance between fusion and fission, and oxidative stress often disrupts this balance. AGEs trigger excessive mitochondrial ROS production and impair the mitochondrial respiratory chain, leading to early‐stage mitochondrial swelling [42]. However, this result needs a further verification in time‐course experiments.

In summary, AGEs contribute to endothelial cell ferroptosis and ferritinophagy in diabetic patients, and ROS clearance can mitigate these processes. This study provides valuable insights into therapeutic strategies for alleviating atherosclerosis in diabetes by targeting ferritinophagy‐dependent ferroptosis due to AGEs accumulation. Although further clinical validation is necessary, our findings lay a strong foundation for considering the alleviation of diabetes‐related atherosclerosis by disrupting the interaction between AGEs and the mechanisms involving ferritinophagy‐dependent ferroptosis.

## Author Contributions

**Yi Huang:** conceptualization (equal), methodology (equal), resources (equal), writing – original draft (equal). **Yongzhi Jin:** writing – original draft (equal). **Junsheng Hu:** formal analysis (equal), investigation (equal), supervision (equal), writing – original draft (equal). **Mengfan Li:** software (equal), validation (equal), writing – review and editing (equal). **Ming Tian:** writing – original draft (equal). **Guang Zeng:** resources (equal), software (equal), visualization (equal), writing – review and editing (equal). **Rong Huang:** conceptualization (equal), methodology (equal), project administration (equal), writing – original draft (equal).

## Ethics Statement

The animal experiment was approved by the Ethical Committee for Animal Research of the Putuo district centre hospital (Putuo Hospital, Shanghai University of Traditional Chinese Medicine) (permit number DWEC‐A‐202309002). The clinical study was approved by the Ethics Committee of the Putuo district centre hospital (Putuo Hospital, Shanghai University of Traditional Chinese Medicine) (permit number PTEC‐R‐2021‐13‐1), and all patients obtained informed consent for pathological examination and signed informed consent.

## Consent

Informed consents were collected from all the patients before this research started. All the patients have agreed to the publication of clinical results.

## Conflicts of Interest

The authors declare no conflicts of interest.

## Data Availability

The raw data that support the findings of this study are available from the corresponding author upon reasonable request.
